# Risk factors for postpartum hemorrhage according to the Robson classification in a low-risk maternity hospital

**DOI:** 10.61622/rbgo/2024rbgo53

**Published:** 2024-06-27

**Authors:** Amanda Botelho, Adriana Luckow Invitti, Rosiane Mattar, David Baptista da Silva Pares, Camilla Parente Salmeron, João Victor Jacomele Caldas, Nathalia Mello, Alberto Borges Peixoto, Edward Araujo Júnior, Sue Yazaki Sun

**Affiliations:** 1 Department of Obstetrics Escola Paulista de Medicina Universidade Federal de São Paulo São Paulo SP Brazil Department of Obstetrics, Escola Paulista de Medicina, Universidade Federal de São Paulo, São Paulo, SP, Brazil.; 2 Department of Gynecology Escola Paulista de Medicina Universidade Federal de São Paulo São Paulo SP Brazil Department of Gynecology, Escola Paulista de Medicina, Universidade Federal de São Paulo, São Paulo, SP, Brazil.; 3 Beneficent Association of Blood Collection São Paulo SP Brazil Beneficent Association of Blood Collection, São Paulo, SP, Brazil.; 4 Hospital Amparo Maternal São Paulo SP Brazil Hospital Amparo Maternal, São Paulo, SP, Brazil.; 5 Gynecology and Obstetrics Service Hospital Universitário Mário Palmério Universidade de Uberaba Uberaba MG Brazil Gynecology and Obstetrics Service, Hospital Universitário Mário Palmério, Universidade de Uberaba, Uberaba, MG, Brazil.; 6 Department of Obstetrics and Gynecology Universidade Federal do Triângulo Mineiro Uberaba MG Brazil Department of Obstetrics and Gynecology, Universidade Federal do Triângulo Mineiro, Uberaba, MG, Brazil.

**Keywords:** Postpartum hemorrhage, Pregnancy, Postpartum period, Risk factors, Robson classification, Hospitals, maternity

## Abstract

**Objective:**

To evaluate the risk factors for postpartum hemorrhage (PPH) according to the Robson Classification in a low-risk maternity hospital.

**Methods:**

We conducted retrospective cohort study by analyzing the medical records of pregnant women attended in a low-risk maternity hospital, during from November 2019 to November 2021. Variables analyzed were: maternal age, type of delivery, birth weight, parity, Robson Classification, and causes of PPH. We compared the occurrence of PPH between pregnant women with spontaneous (Groups 1 and 3) and with induction of labor (2a and 4a). Chi-square and Student t-tests were performed. Variables were compared using binary logistic regression.

**Results:**

There were 11,935 deliveries during the study period. According to Robson’s Classification, 48.2% were classified as 1 and 3 (Group I: 5,750/11,935) and 26.1% as 2a and 4a (Group II: 3,124/11,935). Group II had higher prevalence of PPH than Group I (3.5 vs. 2.7%, p=0.028). Labor induction increased the occurrence of PPH by 18.8% (RR: 1.188, 95% CI: 1.02-1.36, p=0.030). Model including forceps delivery [x^2^(3)=10.6, OR: 7.26, 95%CI: 3.32-15.84, R^2^ Nagelkerke: 0.011, p<0.001] and birth weight [x^2^(4)=59.0, OR: 1.001, 95%CI:1.001-1.001, R^2^ Nagelkerke: 0.033, p<0.001] was the best for predicting PPH in patients classified as Robson 1, 3, 2a, and 4a. Birth weight was poor predictor of PPH (area under ROC curve: 0.612, p<0.001, 95%CI: 0.572-0.653).

**Conclusion:**

Robson Classification 2a and 4a showed the highest rates of postpartum hemorrhage. The model including forceps delivery and birth weight was the best predictor for postpartum hemorrhage in Robson Classification 1, 3, 2a, and 4a.

## Introduction

Postpartum hemorrhage (PPH) is blood loss of more than 500 ml after vaginal delivery or more than 1000 ml after cesarean section in the first 24 hours, or any blood loss from the genital tract that may cause hemodynamic instability. It may be classified as primary or secondary. Primary is postpartum hemorrhage that occurs in the first 24 hours postpartum, and secondary is hemorrhage that occurs from 24 hours to 6 weeks postpartum.^(1)^

Postpartum hemorrhage remains the leading cause of maternal mortality worldwide, accounting for 27% of maternal deaths. Most of these deaths occur in low- and middle-income countries and are associated with limited access to timely and quality care and inadequate availability of resources such as blood products. PPH has become more prevalent due to increasing rates of advanced maternal age, obesity, pre-eclampsia, prolonged labor, caesarean section, induced labor, and multiple pregnancies. In addition, PPH contributes to serious maternal illness, morbidity and permanent disability worldwide. The global prevalence of PPH ranges from 6 to 10%, but varies widely between and within countries.^(2,3)^

In 2001, Michael Robson^(4)^ created the Robson Classification, which allows the prospective identification of clinically relevant groups of pregnant women who differ in cesarean section rates, allowing comparisons within the same institution over time or between institutions. This classification was recommended by the World Health Organization in 2015^(5)^ for global use to assess cesarean section rates worldwide. It is based on six obstetric concepts: parity (nullipara, multipara), previous cesarean (yes, no), onset of labor (spontaneous, induced, cesarean before labor), gestational age (term, preterm), fetal presentation (cephalic, breech, transverse), number of fetuses (single, multiple).^(4)^

In Brazilian teaching hospitals, mean cesarean section rates ranged from 24.8% to 75.1%, far exceeding recommended values, even in Robson groups considered low risk for cesarean section (groups 1 to 4).^(6)^ In a Swedish study, the authors examined the trends of PPH according to the Robson classification in deliveries between 2000 and 2016. PPH rates varied between the Robson Classification groups, ranging from 4.5% in group 3 to 14.3% in group 4b. Increasing trends in PPH were seen in all Robson Classification groups except groups 2b and 4b (prelabor cesarean section).^(7)^To the best of our knowledge, no previous studies have evaluated the factors associated with PPH according to the Robson Classification in a Brazilian population.

The objective of this study was to evaluate the risk factors for PPH according to the Robson Classification in a low-risk maternity hospital in the city of São Paulo, Brazil.

## Methods

We conducted a retrospective cohort study by analyzing the medical records of women who delivered at the Hospital, which serves low-risk obstetric pregnant women in the city of São Paulo, Brazil, from November 2019 to November 2021. The population consisted of pregnant women divided into two groups according to the Robson classification: Group I (Robson 1 - nulliparous, single cephalic, ≥ 37 weeks, spontaneous labor; and Robson 3 - multiparous, single cephalic, ≥ 37 weeks, spontaneous labor) and Group II (Robson 2a - nulliparous, single cephalic, ≥ 37 weeks, induced labor; and Robson 4a - multiparous, single cephalic, ≥ 37 weeks, induced labor).^(4)^

Inclusion criteria were as follows: 1) low-risk singleton pregnancies, 2) fetus in cephalic presentation, 3) gestational age ≥ 37 weeks as calculated by last menstrual period and confirmed by first-trimester ultrasound, 4) admitted for induction of labor or in the active stage of labor.

Patient data were retrieved from three databases at Hospital: the maternity record book, the pharmacy drug dispensing list, and the blood transfusion record from the blood bank. After the initial collection, the data were cross-checked with the PPH registration chart, which is completed monthly by the maternity hospital’s obstetric team.

In the birth registration book of the normal delivery center of the maternity hospital, which is filled in by nurses, the occurrence of PPH is included among the delivery data. The pharmacy drug dispensing lists and blood bank records showed the amount of oxytocin, ergotamine, misoprostol, and tranexamic acid, respectively, and blood transfusions used for each patient.

According to local protocol, labor induction was performed with 25 µg tablets of misoprostol in pregnant women with Bishop scores < 6. The drug was inserted into the posterior vaginal fornix at a dosage of 1 tablet every six hours for a maximum of 24 hours (100 µg = 4 tablets). In cases of Bishop scores ≥ 6 induction of labor was performed using oxytocin through a continuous intravenous infusion at an initial rate of 0.12 U/h and increased to a maximum of 1.2 U/h. Induction of labor was considered unsuccessful after 4 tablets of misoprostol were inserted into the vagina of a pregnant woman or after a total of 7.2 U oxytocin intravenous without cervical changes.

In our institution, pregnant women were classified according to their risk of PPH at the time of admission to the labor ward. It was considered low risk for PPH pregnant women with < 4 previous vaginal deliveries, singleton pregnancies, no previous uterine scars, no previous PPH, no known bleeding disorder. It was considered medium risk for PPH pregnant women with previous cesarean sections or previous myomectomy, ≥ 4 vaginal deliveries, chorioamnionitis, gestational hypertension, multiple pregnancies, estimated fetal weight > 4,000 grams, history of PPH, and severe obesity (body mass index - BMI > 35 kg/m^2^). Pregnant women with placenta previa, suspected placenta accreta, abruptio placentae, and coagulopathy were considered high risk for PPH.^(8)^In addition, pregnant women with ≥ 2 medium risk factors were considered as high-risk to PPH.^(9)^

We considered PPH when there was a blood loss of 500 ml or more after vaginal delivery or 1000 ml or more after cesarean section associated with a shock index (heart rate/systolic blood pressure ratio) ≥ 0.9, according to the “Zero Maternal Death from Postpartum Hemorrhage”.^(9)^ According to the local protocol, blood loss was quantified by gravimetry. After delivery, the total weight of bloody gauze pads was measured and subtracted from the known weight of them when dry. The difference in weight between wet and dry in grams approximates the volume of blood in milliliters.^(10,11)^

The following variables were assessed: maternal age, number of previous pregnancies, parity, gestational age, time between deliveries (inter partum time), risk assessment of PPH at admission, number of misoprostol tablets during induction of labor, number of oxytocin units during induction of labor, type of delivery, controlled cord clamping, prophylactic use of postpartum oxytocin, uterine laceration, uterine atony, need for hemostatic suture of B-Lynch, need for use of Bakri balloon, postpartum hysterectomy, estimated volume of blood loss, higher indication of shock observed, use of oxytocin for PPH treatment, use of ergotamine for PPH treatment, use of tranexamic acid for PPH treatment, transfusion of blood products, birth weight, APGAR scores at 1st and 5th minute, need for admission to neonatal intensive care unit, maternal death and neonatal death.

Data were analyzed using SPSS version 20.0 software (SPSS Inc, Chicago, IL, USA). Quantitative variables were subjected to the Kolmogorov-Smirnov test for normality and presented as means and standard deviations. Categorical variables were described as absolute and percentage frequencies and presented in tables and graphs. Differences between categorical variables and their proportions were analyzed using the chi-squared test. The effect of groups on continuous variables was analyzed using the Student-t test (parametric distribution) or the Mann-Whitney test (non-parametric distribution). Binary logistic regression was used to determine the best predictors of PPH. The odds ratio (OR) for the development of PPH with statistical difference between groups was determined by stepwise binomial logistic regression. Receiver operating characteristics (ROC) curve for determination of the best birth weight to predict postpartum hemorrhage in pregnant women with gestational age ≥ 37 weeks admitted to induction or active phase of labor. The significance level for all tests was p < 0.05.

This study was approved by the Local Ethics Committee 5.054.866 (CAAE: 50490821.7.0000.5505).

## Results

**Figure 1:**
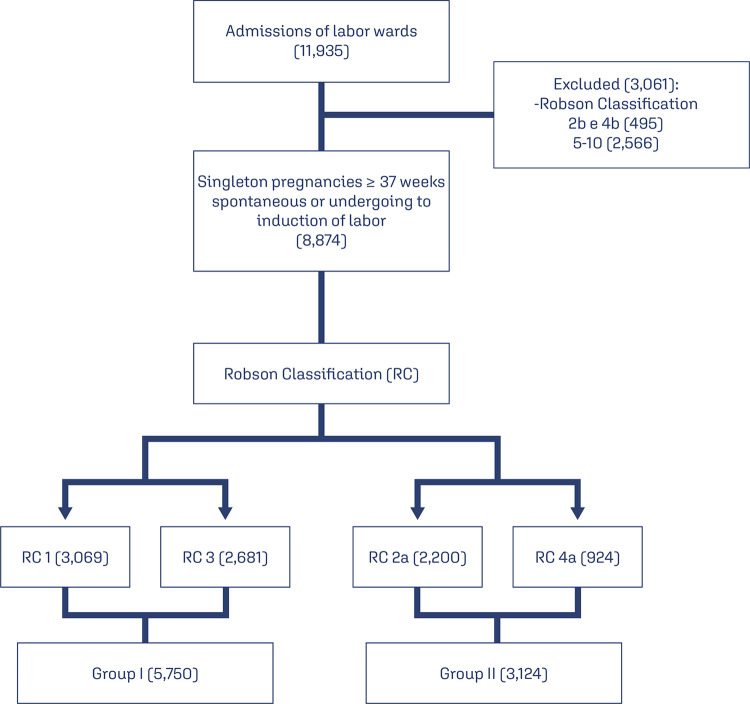
Selection of the patients included in the study

From November 2019 to November 2021, 11,935 deliveries were performed at the Amparo Maternal Hospital. At the time of admission to the labor ward, according to Robson’s classification, 48.2% of pregnancies were admitted in classifications 1 and 3 (5,750/11,935), 26.1% in classifications 2a and 4a (3,124/11,935), 4.2% in classifications 2b and 4b (495/11,935), and 21.5% in classifications 5 to 10 (2,566/11,935). For the final statistical analysis, 8,874 pregnant women were considered, divided into two groups: Group I - classifications 1 and 3, and Group II - classifications 2a and 4a (Figure 1).

The characteristics of the study population are shown in table 1. There was significant differences between the groups regarding to maternal age (p=0.010), number of previous pregnancies (p=0.043), gestational age at admission to the labor ward (p<0.001), birth weight (p<0.001), parous status (p<0.001), use of misoprostol during labor induction (p<0.001), use of oxytocin during labor induction and/or conduction (p<0.001), vaginal delivery (p<0.0001), forceps delivery (p=0.026), cesarean section (p<0.0001), and controlled umbilical cord traction (p<0.001).

**Table 1:** Clinical characteristics of study population

Variables	Group I (5,750)	Group II (3,124)	p-value
Age (years)	25.6 (6.2)	26.0 (5.9)	0.010 ^†^
Number of pregnancies	2.0 (0.0-10.0)	1.0 (1.0-9.0)	0.043 ^∫^
Previous delivery			<0.001 ^§^
Nulliparous	53,1% (3,053/5,750)	66,1% (2,085/3,124)	
Multiparous	46.9% (2,697-5,750)	33,3% (1,039-3,124)	
Gestational age (weeks)	39.4 (37.4-43.6)	40.4 (37.4-42.6)	<0.001 ^∫^
Hemorrhagic risk assessment admission			
High	2.5% (2/79)	1.4% (1/73)	>0.999 ^§^
Medium	5.1% (4/79)	11.0% (8/73)	0.233 ^§^
Low	92.4% (73/79)	87.7% (64/73)	0.417 ^§^
Use of misoprostol for labor induction	0.0% (0-5,750)	73.9% (2,310-3,124)	<0.001 ^§^
Use of oxytocin for labor induction and/or conduction	9.8% (5,63/5,750)	28.0% (8,76/3,124)	<0.001 ^§^
Type of delivery			
Vaginal	91.7% (5,272/5,750)	62.7% (1,958/3,124)	<0.0001 ^§^
Forceps	0.4% (25/5,750)	0.8% (26/3,124)	0.026 ^§^
Cesarean section	7.9% (4,53/5,750)	36.5% (1,140/3,124)	<0.0001 ^§^
Draw controlled cord	92.6% (5,326/5,750)	68.1% (2,128/3,124)	<0.001 ^§^
Prophylactic oxytocin	99.9% (5,743/5,750)	100.0% (3,124/3,124)	0.974 ^§^
Birth weight (grams)	3,255.0 (1,035.0-4,850.0)	3,330.0 (1,915.0-5,470.0)	<0.001 ^∫^
Macrosomia	3.6% (204/5,711)	5.8% (181/3,097	<0.001 ^§^
APGAR score at the 1st min	9.0 (0.0-10.0)	9.0 (0.0-10.0)	0.921 ^∫^
APGAR score at the 5th min	9.0 (0.0-10.0)	9.0 (0.0-10.0)	0.897 ^∫^

Group I: Robson classification 1 and 3; Group II: Robson classification 2a and 4a; Student-t test ^†^: mean (standard deviation); Mann-Whitney ^∫^: median (minimum-maximum); Chi-square ^§^: percentage (n/N); p<0.05

There was a significant difference in the prevalence of PPH between the groups (p=0.028). Patients classified as Robson 2a and 4a had higher prevalence of PPH than patients in classes 1 and 3 (3.5 vs. 2.7%, p=0.028). The group that underwent labor induction (Group II) had 32.3% higher odds of experiencing PPH than the non-induced group (Group I) (OR: 1.323, 95% CI: 1.03-1.69, p=0.030). Labor induction may increase the occurrence of PPH by 18.8% (RR: 1.188, 95% CI: 1.02-1.36, p=0.030). A significant difference between the groups was observed in the volume of blood lost during delivery (p<0.001). Patients of Group II had a higher median blood loss than those of Group I (250.0 vs. 200.0 ml, p<0.001). A higher prevalence of oxytocin use (2.5% vs. 1.5%, p<0.001) and ergotamine use (2.3% vs. 1.7%, p=0.039) was observed in Group II compared to those of Group I (Table 2).

**Table 2:** Postpartum hemorrhage, causes and treatment, in pregnant women with gestational age ≥ 37 weeks admitted for induction or active phase of labor

Variables	Group I (5,750)	Group II (3,124)	p-value
Postpartum hemorrhage	2.7% (153/5,750)	3.5% (109/3,124)	0.028 ^§^
Shock index ≥ 0.9	1.0% (56/5,747)	1.0% (32/3,119)	0.815 ^§^
Causes of postpartum hemorrhage			
Uterine atony	1.4% (83/5,750)	1.9% (60/3,124)	0.088 ^§^
Laceration path	0.3% (15/5,750)	0.4% (13/3,124)	0.213 ^§^
Other	1.0% (58/5,750)	1.1% (33/3,124)	0.826 ^§^
Volume of blood loss (ml)	200.0 (5-1,830)	250 (5-2,585)	< 0.001 ^∫^
PPH treatment			
Oxytocin	1.5% (85/5,749)	2.5% (79/3,124	< 0.001 ^§^
Ergotamine	1.7% (95/5,750)	2.3% (71/3,124	0.039 ^§^
Misoprostol	0.9% (49/5,749)	1.3% (40/3,124)	0.053 ^§^
Tranexamic acid	1.6% (94/5,750)	2.1% (67/3,124)	0.086 ^§^
B-Lynch hemostatic suture	0.0% (1/5,743)	0.0% (1/3,119)	0.661 ^§^
Bakri balloon	0.0% (0/5,743)	0.1% (2/3,119)	0.055 ^§^
Hysterectomy	0.0% (0/5,743)	0.0% (1/3,119)	

Group I: Robson classification 1 and 3; Group II: Robson classification 2a and 4a; PPH: postpartum hemorrhage; Student-t test ^†^: mean (standard deviation); Mann-Whitney ^∫^: median (minimum-maximum); Chi-square ^§^: percentage (n/N); p<0.05.

**Figure 2:**
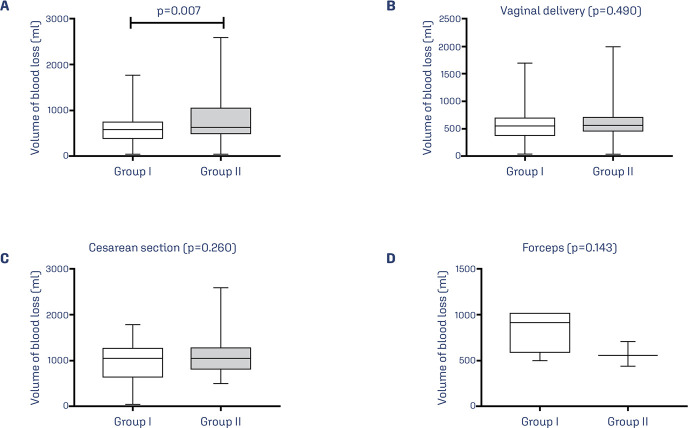
Volume of blood loss in patients with gestational age ≥ 37 weeks’ gestation classified as Robson 1 and 3 (Group I) and Robson 2a to 4a (Group II) with postpartum hemorrhage. (A) Volume of blood loss among all patients in Group I and Group II; (B) Volume of blood loss during vaginal delivery among patients in Group I and Group II; (C) Volume of blood loss during cesarean delivery among patients in Group I and Group II; (D) Volume of blood loss during forceps delivery among patients in Group I and Group II

Considering only patients with PPH, Group II had higher median volume of bleeding compared to patients in Group I [580.0 (400.0-742,5) vs. 610.0 (500.0-610) ml, p=0.007] (Figure 2A). Considering only the patients with PPH in Group I, 86.3% (132/153), 10.4% (16/153), and 3.3% (5/153) underwent vaginal delivery, cesarean section, and forceps delivery, respectively. Considering only the patients with PPH in Group II, 66.0% (72/109), 30.3% (33/109), and 3.7% (4/109) underwent vaginal delivery, cesarean section, and forceps delivery, respectively. Considering only patients with PPH, no significant differences between the groups on bleeding volume was observed in patients who underwent vaginal delivery (p=0.490) (Figure 2B), cesarean section (p=0.260) (Figure 2C), and forceps delivery (p=0.143) (Figure 2D).

Considering all the cases included in the study, significant association was observed between presence of PPH and induction of labor (p=0.030), birth weight ≥ 4000 grams (p<0.0001), and forceps delivery (p<0.0001). No significant association was observed between PPH and vaginal delivery (p=0.125) and cesarean section (p=0.743). Patients with PPH had higher prevalence of labor induction (41.6% vs 35.0%, p=0.030), birth weight ≥ 4000 grams (14.4% vs 4.1%, p<0.0001), and forceps delivery (3.4% vs 0.5%, p<0.0001) (Table 3).

**Table 3:** Association between postpartum hemorrhage and induction of labor, birth weight, and type of delivery

	PPH	Absence PPH	OR (95%CI)	p-value
Induction of labor	41.6% (109/262)	35.0% (3,015/8,612)	1.32 (1.03-1.66)	0.030 ^§^
Birth weight	3,345 (2,295-4,645)	3275 (1,035-5,470)		<0.0001 ^∫^
Birth weight ≥ 4000 grams	14.4% (31/215)	4.1% (353/8,589)	3.91 (2.64-5.84)	<0.0001 ^§^
Type of delivery				
Vaginal	77.9% (204/262)	81.6% (7,026/8,612)	0.79 (0.60-1.06)	0.125 ^§^
Forceps	3.4% (9/262)	0.5% (42/8,612)	7.25 (3.54-15.08)	<0.0001 ^§^
Cesarean section	18.7% (49/262)	17.9% (1,544/8,612)	1.05 (0.76-1.45)	0.743 ^§^

PPH: postpartum hemorrhage; OR: Odds Ratio; CI: confidence interval. Mann-Whitney ∫: median (minimum-maximum); Qui-Quadrado §: percentage (n/N), p<0.05

A stepwise binary logistic regression model was constructed using induction of labor, birth weight ≥ 4000 grams, and type of delivery to evaluate the best predictors of PPH. It was observed that induction of labor lost its predictive ability for PPH [x^2^(1) =4.73, OR: 1.24, 95%CI: 0.971-1.607, R^2^ Nagelkerke: 0.002, p=0.083] when type of delivery and birth weight were added to the model. The model including forceps delivery [x^2^(3)=47.4, OR: 7.19, 95%CI: 3.444-15.030, R^2^ Nagelkerke: 0.011, p<0.001] and birth weight ≥ 4000 grams [x^2^(4)=29.09, OR: 3.07, 95%CI:2.078-4.554, R^2^ Nagelkerke: 0.014, p<0.001] was the best model for predicting PPH in patients classified as Robinson 1, 3, 2a, and 4a (Table 4).

**Table 4:** Adjusted risk of postpartum hemorrhage in pregnant women with gestational age ≥ 37 weeks using induction of labor, birth weight, and type of delivery

Variables	aOR	95% CI	p-value
Robinson Classification 2A and 4A	1.24	0.971-1.607	0.083
Type of delivery			<0.001
Cesarean section	1.003	0.635-1.345	0.873
Forceps delivery	7.19	3,444-15,030	<0.001
Birth weight	3.07	2,078-4,554	<0.001

aOR: adjusted odds ratio; CI: confidence interval; Stepwise Binary logistic regression. p<0.05

**Figure 3:**
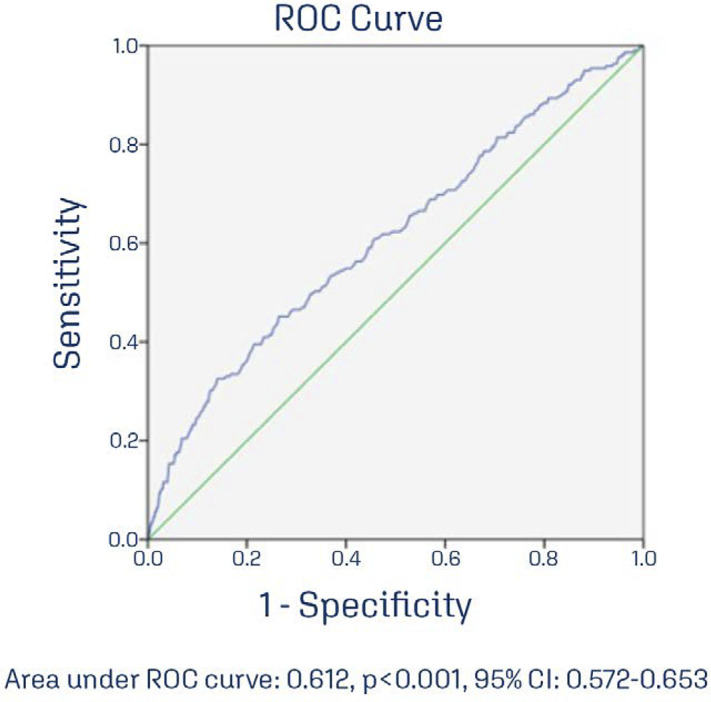
Receiver operating characteristics curve for determination of the best birth weight to predict postpartum hemorrhage in pregnant women with gestational age ≥ 37 weeks admitted to induction or active phase of labor

The ROC curve was used to determine the best sensitivity and the best cutoff value for birth weight to predict PPH (Figure 3).

Birth weight was a poor predictor of PPH. A birth weight of 4,255 grams was able to correctly identify 37.0% of patients with PPH with a false positive rate of 10%. A birth weight of 4,132 grams was able to correctly identify 74.0% of patients who had PPH with a false positive rate of 21%.

## Discussion

In our study, in a low-risk maternity hospital, most patients admitted to the delivery room were classified in Robson groups 1, 2a, 3 and 4a. A total of 11,774,665 live births were reported in Brazil during 2014 to 2017 from the Brazilian Live Birth Information System. According to the Robson Classification, the groups 1 to 4 accounted for 60.2% of live births and 47.1% of all cesarean sections.^(12)^

In a study conducted in a maternity hospital in Honduras, using Robson’s 10 classification groups to analyze cesarean rates, Groups 1 and 3 with 26.6% (291/1,136) and 13.5% (153/1,136), respectively, were the second and third larger contributors to the cesarean sections. Groups 2a and 4a had high induction success, with low cesarean section rates (18.4 and 16.9%, respectively).^(13)^

Postpartum hemorrhage is a serious obstetric complication that remains the leading cause of maternal death worldwide.^(14)^It can be clinically defined and diagnosed as excessive bleeding that renders the patient symptomatic (blurred vision, dizziness, or syncope) and/or results in signs of hypovolemia (hypotension, tachycardia, and oliguria).^(15)^The most traditional way of conceptualizing postpartum hemorrhage is as blood loss greater than 500 ml for vaginal delivery and 1000 ml for cesarean section,^(16)^but this definition cannot be palpable and tends to underestimate the volume of blood lost by patients.^(17)^ It should be noted that while gravimetric measurement may not be flawless for defining a case of PPH, it is theoretically the most tangible “palpable” method of measuring blood loss in clinical practice, because clinical signs are usually indicative of a more advanced stage of blood loss. Borovac-Pinheiro et al.^(18)^used the gravimetric method (sum of the volume collected from the drape with the weight of gauzes, compresses and pads - subtracting the dry weight) to estimate the total blood loss after delivery.

It is important to highlight that the institutional protocol associates the shock index ≥ 0.9 in the diagnosis of PPH avoiding under or overdiagnosis of PPH based only in the volume blood loss. The main causes of PPH are uterine atony, lacerations in the birth canal, retained placental fragments and coagulation disorders, macrosomia, twin pregnancies, polyhydramnios, use of tocolytics, halogenated anesthetics, chorioamnionitis, and cesarean section.^(9)^Other risk factors for PPH have also been felt such as obesity and multiparity, and tachytocyte deliveries.^(19)^It should be noted that many women with PPH do not have classically identified risk factors.^(19)^One study showed an association between induction of labor and PPH in low-risk parturients comparing 4,450 women with PPH and 1,744 controls.^(20)^After adjustment for all potential confounders, labor induction was associated with a significantly higher risk of PPH for both oxytocin and prostaglandins. In our study, also using a low-risk population, patients in Group II (Robson classification 2a and 4a) had higher rates of PPH than patients in Group I (Robson classification 1 and 3). These differences may be explained by the labor induction itself, oxytocin use for induction and/or conduction, and cesarean section in Group II than in Group I. In a Swedish study, the rates of PPH varied between Robson Classification, ranging from 4.5% in Group 3 to 14.3% in Group 4b between 2000 and 2016. Rates of PPH increased significantly over time in Groups 1, 2a, 4a and 5, but not in groups 2b (nulliparous with previous cesarean section) or 4b (multiparous with previous cesarean section). Group I - Robson Classification 1 and 3 presented 6.9 and 4.5%, respectively, and Group II - Robson Classification 2a and 4a presented 11.0 and 5.6%, respectively, of PPH.^(7)^ These results are consistent with ours showing that patients with induced labor had more PPH than patients with spontaneous labor.

In a published study, 666 cases of PPH were evaluated and compared with 645 controls, the obstetric risk factors significantly associated with primary hemorrhage, in descending order and taking into account the relative risks, were placental retention, prolonged labor, placental accretism, cervix laceration, instrumental delivery, fetal macrosomia, hypertensive disorders and induction of labor with oxytocin.^(21)^In our study, the model including forceps and birth weight was the best model to predict PPH in patients classified as Robson 1, 3, 2a and 4a.^(18)^Episiotomy, longer second stage of labor and forceps delivery were related to blood loss > 500 ml within 2 hours, in the univariate analysis. However, in the multivariate analysis, only forceps remained associated with bleeding > 500 ml within 2 hours.

In our study, birth weight was a poor predictor of PPH (Area under ROC curve: 0.612, 95% CI: 0.572-0.653). A study evaluated the usability of the relationship between birth weight and placental weight [fetoplacental ratio (FPR)] in predicting PPH and neonatal intensive care unit (NICU).^(22)^These authors assessed 812 women, being 7% with PPH. The FPR was found as an independent predictor for PPH by nearly 3.5-fold and women who experienced PPH had heavier placenta and lower FPR.

Women with a previous episode of PPH have a 15% risk of recurrence in a subsequent pregnancy.^15^ Measures to prevent PPH should be incorporated into the routine of all professionals assisting patients in labor. Postpartum oxytocin is the most important intervention to prevent PPH, as it can reduce more than 50% of cases of hemorrhage due to uterine atony.^(9)^Active management in the third stage of labor also reduces the risk of excessive maternal blood loss; in addition to oxytocin shortly after birth, timely umbilical cord clamping, controlled cord traction, uterine massage, and skin-to-skin contact are other effective measures in preventing early hemorrhage.^(23)^

The strength of our study was a cohort of low-risk pregnancies from a single reference center with a standard protocol for the risk of PPH. The weakness was the relatively small sample size.

## Conclusion

In summary, to the best of our knowledge the present study was only the second to use the Robson Classification groups to identify risk factors for postpartum hemorrhage. Robson Classification 2a and 4a showed the highest rates of postpartum hemorrhage. The model including forceps delivery and birth weight was the best predictor for postpartum hemorrhage in Robson Classification 1, 3, 2a, and 4a.
